# Early‐Life Policies and the Prevention of Multiple Long‐Term Conditions: Findings From a Stakeholder Workshop Developed Collaboratively With Public Contributors

**DOI:** 10.1111/hex.70928

**Published:** 2026-10-16

**Authors:** Sebastian Stannard, Nida Ziauddeen, Robin Poole, I. Cook, Emilia Holland, Erin Panes, Reet Nagra, Jack Welch, Simon DS Fraser, Nireen A Alwan

**Affiliations:** ^1^ School of Primary Care, Population Sciences and Medical Education, Faculty of Medicine, University of Southampton Southampton UK; ^2^ NIHR Applied Research Collaboration Wessex Southampton UK; ^3^ NIHR Southampton Biomedical Research Centre University of Southampton and University Hospital Southampton NHS Foundation Trust Southampton UK; ^4^ Southampton City Council Southampton UK; ^5^ Public Policy, Southampton, University of Southampton Southampton UK; ^6^ University of Southampton Southampton UK; ^7^ University Hospital Southampton NHS Foundation Trust Southampton UK

**Keywords:** co‐production, early‐life, public contributors, stakeholders, workshop

## Abstract

**Introduction:**

The multidisciplinary ecosystem to study life course determinants and prevention of early‐onset burdensome multimorbidity (MELD‐B) project examined early‐life factors that contribute to the risk of developing multiple long‐term conditions (MLTC) in adulthood. Insights from an initial stakeholder workshop shaped the project's direction and priorities. To continue this collaborative approach, we developed a second workshop with public contributors to reflect on how our research aligns with current early‐life policies. We defined these as policies that support children and their families at an early stage to mitigate risk factors and strengthen protective factors for health and wellbeing across the life course, thereby reducing the risk of MLTC later in life. The workshop explored challenges facing children and young people within the system, considered practical applications of the research findings and identified priorities for future research.

**Methods:**

The research team worked with three public contributors (one adult member of the MELD‐B Advisory Group and two young researchers) to develop the workshop in terms of its questions, structure and format. Stakeholders were divided into three mixed‐background groups, each facilitated by two team members, and discussions centred on predefined questions. Padlet (an online whiteboard) was used to collate ideas. Themes from the breakout rooms and full group discussions were initially identified with the aid of AI (Institutional account of Microsoft Copilot).

**Results:**

Fifteen stakeholders from backgrounds including health visiting (*n* = 2), charities (*n* = 1), academics (*n* = 3), healthcare practitioners (*n* = 5) and council employees (*n* = 4), attended the workshop. Stakeholders emphasised that wider systems and environments shape child and family outcomes, with poverty and socioeconomic inequality acting as major upstream drivers. Stakeholders noted that gaps in data, fragmented information systems and poor linkage hinder informed decision‐making, while short‐term political and budget cycles, workforce pressures, insufficient funding and implementation challenges reduce the impact of early‐years policies. Stakeholders agreed that the early years are the most critical period for effective action and require coordinated, cross‐sector, whole‐system approaches. Stakeholders highlighted the need for research to be translated into clear, actionable guidance, and stressed the importance of participatory engagement, clear communication and relational ways of working.

**Conclusions:**

Our workshop identified key barriers to early‐life policies as well as areas of best practice to guide future action.

**Patient or Public Contribution:**

A public contributor who is a member of the MELD‐B PPI Advisory Group and two young researchers from NxtGen, a young researcher training programme at the University of Southampton, were actively involved throughout the development and delivery of the workshop. They helped identify stakeholders to invite, developed the workshop agenda and contributed to the design of presentation materials. Their input shaped how key messages were communicated, helping to ensure that these were engaging, accessible and aligned with policies relevant to young people identified through previous NxtGen research. They also advised on the choice of interactive tools, with Padlet selected after testing alternative options. The public contributor attended the workshop, facilitated one of the breakout rooms and participated in discussions. All three contributed to the manuscript, including writing and editing the sections describing their involvement, providing reflective statements and designing the figure. All three are named co‐authors.

## 1 Introduction

The multidisciplinary ecosystem to study life course determinants and prevention of early‐onset burdensome multimorbidity (MELD‐B) project [1], which ran from June 2022 to March 2026, aimed to identify early‐life targets up to age 18 for the prevention of early onset (by age 65), burdensome multimorbidity (also known as multiple long‐term conditions [MLTC]), and to characterise the concept of ‘burden' for people living with MLTC.

As part of this project, the research team undertook substantial conceptual and methodological development to consider how the early‐life course should be understood and analysed in relation to the development of MLTC in adulthood [2, 3]. This work resulted in a co‐produced conceptual framework describing the early‐life domains that may influence future MLTC risk (Figure 1) [2]. The framework was developed through a review of existing research evidence and policy alongside consultation with public contributors through two workshops. It identified 12 interconnected domains, including adverse childhood experiences, socioeconomic circumstances, education, and social and physical environmental factors, reflecting the complex pathways through which early‐life experiences may shape later‐life health. This framework subsequently informed the empirical and modelling work undertaken across the MELD‐B programme [4, 5, 6, 7]. This included methodologies to first analyse competing early‐life domains of risk for the development of MLTC [4, 5, 6], and secondly to explore the impact of real‐world early‐life interventions on reducing the likelihood of MLTC later in life [7].

**Figure 1:**
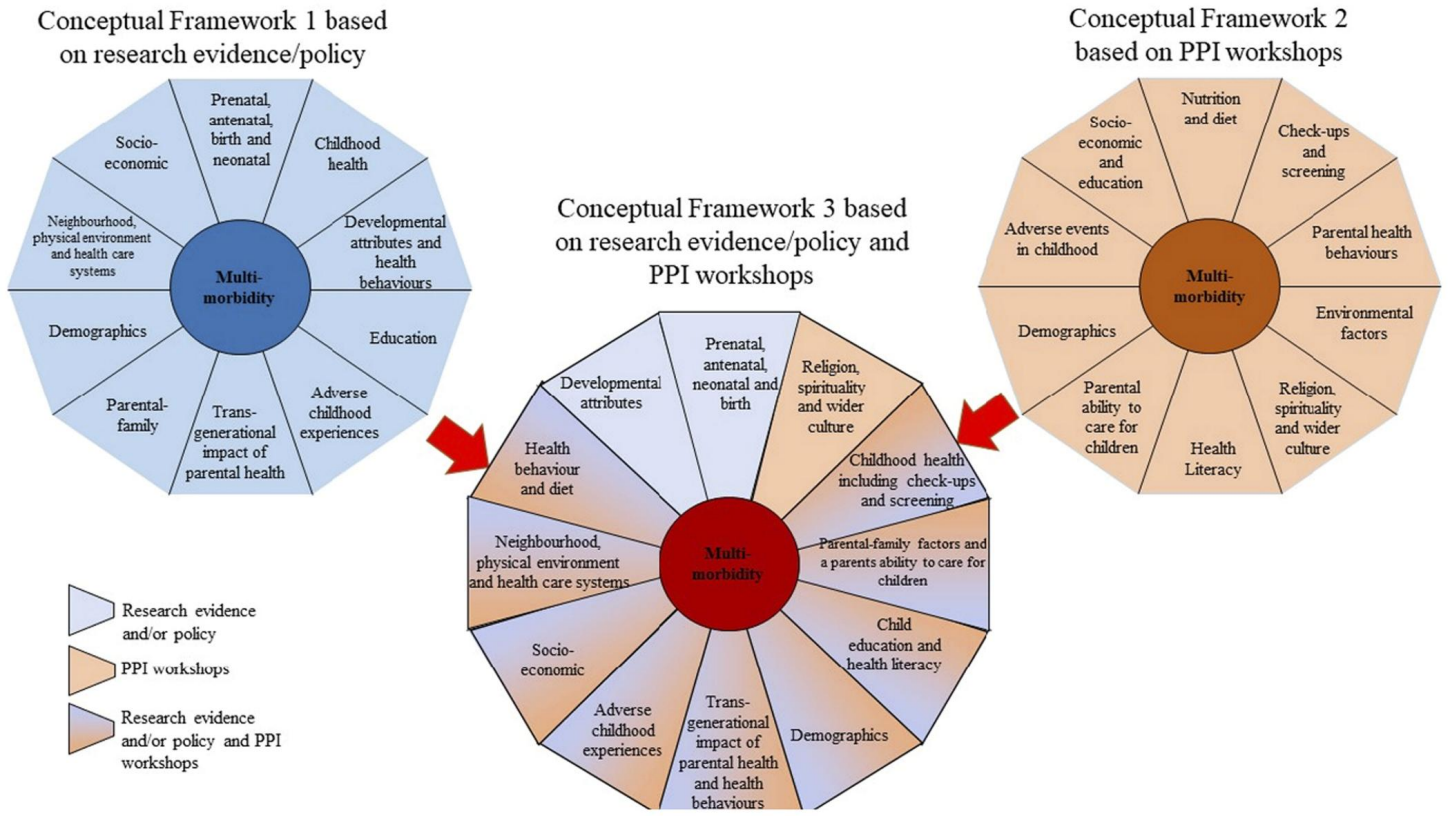
The development of a conceptual framework for characterising life course determinants of MLTCs. Reproduced from Stannard et al. © The Authors 2023. Licensed under CC BY 4.0 [2].

It is important that large research projects, such as MELD‐B conduct research with Patient and Public Involvement and Engagement (PPIE). The NIHR defines co‐production as ‘an approach where researchers, practitioners and members of the public work together, sharing power and responsibility from the start to the end of the project, including the generation of knowledge’ [8]. PPIE involvement is vital for enhancing the quality, outcomes and applicability of research [9]. When members of the public collaboration with stakeholder engagement activities, they help ensure the research remains rooted in lived experience and retains a person‐centred focus [10].

PPIE has been integral throughout the MELD‐B project. We have collaborated closely with members of the public and a range of stakeholders. This included an embedded PPI advisory board within MELD‐B and the co‐production of a conceptual framework achieved through two focus groups: one with young adults (aged 18–30) regardless of MLTC status, and the second with older adults (aged 40–65) living with self‐reported MLTC [2]. In total, 20 public contributors from a range of demographic backgrounds (age, gender, ethnicity and geographical location) [2] helped to design the conceptual framework.

More recently, we conducted a stakeholder workshop in 2023, developed collaboratively with public contributors, to inform the next steps of the MELD‐B project's early prevention workstream [11]. Discussions focused on childhood mental health, educational attainment, early identification of health conditions and neurodiversity, nutritional choices, transitional periods, the virtual world and intermediate outcomes on the pathway to future ill health [11]. Stakeholders suggested that family‐targeted interventions were important in early childhood, whereas individually focused interventions might become more important at secondary‐school age. They also identified birth, ages 5–7 and ages 10–11 as important intervention points because these periods often coincide with transitions such as school entry and movement from primary to secondary school [11].

Given that feedback from the previous workshop has been integrated into research conducted across the MELD‐B project, we felt it was important to take a further collaborative approach. We therefore collaborated with members of the public to run a second stakeholder workshop to highlight how stakeholder perspectives had shaped the project, and to enable discussion about how our findings fit within the political and policy landscape. For example, recent years have seen several major policy developments relevant to children and young people's health and wellbeing in the UK. These include the Online Safety Act [12], which aims to create safer online environments and reduce exposure to harmful online content. The Children's Wellbeing and Schools Bill [13], which introduces reforms relating to schools, safeguarding and children's services in England. Legislation relating to tobacco, vaping and the sale of high‐caffeine energy drinks [14, 15], which seeks to reduce exposure to behaviours associated with adverse health outcomes. Wider socioeconomic policies, such as the removal of the two‐child benefit limit, may also influence child health through their effects on family poverty and financial security [16]. Alongside the introduction of new policies, there has been scaling back of earlier early‐years programmes, such as Sure Start, which have previously been associated with improvements in child outcomes [17].

Within the second workshop, we wanted to explore the challenges facing children and young people within this political and policy context, how our research might be used in practice, and to identify priorities for future research. The aims of this second stakeholder workshop were therefore to:

1. Reflect on how findings from the previous workshop have been integrated into the MELD‐B project.
2. Present our findings from MELD‐B to demonstrate how our research explored the long‐term health impacts of early‐life interventions.
3. Identify priority early‐life policies that can support and improve children's health.
4. Explore how current and future policies may influence young people's health across the life course.
5. Examine how policy and research evidence can be connected to reduce life course health inequalities.

This paper discusses how the stakeholder workshop was developed in collaboration with public contributors, the themes that emerged from the workshop, and their relevance to policy and practice.

## 2 Methods

### 2.1 Collaborative Involvement of Public Contributors in Workshop Development

Three public contributors (one adult member of the MELD‐B Advisory Group and two young researchers) played a key role in shaping the workshop. The public contributor was a member of the wider MELD‐B PPIE advisory board, which oversees the project. The young researchers, aged between 15 and 18, were identified because they had previously engaged with NxtGen (formerly the Young Researcher Training Programme) [18] at the University of Southampton. Because the workshop focused on early‐life issues and policies, involving young people in its development was important to ensure that their perspectives informed the workshop questions, structure and design.

Together with the three public contributors, we identified stakeholders to invite and collaboratively developed the workshop agenda, including its structure, format and content. The public contributors identified three areas that they felt should frame the workshop: (1) the current policy context affecting children and young people, (2) early‐life policies that matter most to children and young people, drawing on insights from previous NxtGen work, and (3) health inequalities and their importance in shaping early‐life outcomes. The public contributors worked with the research team to develop agenda items around each topic, helping to ground discussions in the perspectives and priorities of children and young people. They also helped to design visual approaches for communicating concepts such as health inequalities to stakeholders.

Alongside setting the workshop agenda, the public contributors led decisions about the most effective interactive tools for stakeholder engagement and information capture. Following testing with the public contributors, Padlet Inc. [19] was selected because it was easier to use than Microsoft Teams Whiteboard and enabled participants to contribute virtual post‐it notes, including anonymously, while facilitating the organisation and export of workshop outputs. The public contributors also contributed to the design of the PowerPoint slides used during the workshop, helping to streamline content and promote more engaging, less text‐heavy communication of key messages.

The three public contributors were invited to attend the workshop and to participate in both full‐group and breakout discussions. Although the young researchers were unable to attend due to their educational commitments, one public contributor did take part and supported facilitation during the workshop. This included contributing to discussions, assisting in the second breakout room by taking notes and providing feedback during the wider group discussions. Following the workshop, the public contributors were involved in the write‐up of this paper, particularly supporting the drafting and editing of the co‐production sections, contributing to the figure presented in the paper and providing reflective statements about their roles on the project. The three public contributors were invited to be co‐authors on the paper.

We recognised that power differences existed between academic researchers and public contributors, particularly the young researchers from NxtGen. As part of our approach to collaborative involvement, we sought to create an inclusive and supportive environment in which all public contributors could participate meaningfully. Meeting arrangements were flexible and agreed collaboratively, with meetings scheduled around public contributors' availability. Workshop materials and draft documents were circulated in advance to allow sufficient time for review and reflection. Meetings were informal and conversational, and materials were reviewed and revised iteratively so that public contributors could comment on, amend and refine the workshop content. These practices were intended to reduce, rather than eliminate, power imbalances and support public contributors' involvement in decisions about stakeholder selection, workshop design, facilitation, interpretation of findings and manuscript preparation. Academic researchers actively encouraged all contributors to share their views and treated their contributions as equally important to those of the research team. Nevertheless, responsibility for the overall research project remained with the academic team.

#### 2.1.1 Public Contributors Reflective Statements

Reflective statement 1: ‘I was pleased to support the planning and facilitation of this workshop. Working with young people who were also trained in research and could meaningfully contribute to issues important to their generation, and who were seeking to change life outcomes as part of their development, made it all the more impactful. I enjoyed having a central role to both participate and facilitate the discussions on the day with experts across different fields, where I was treated as being among equals and not just ‘the PPI’ in the room. I look forward to seeing what can happen as a result of the changes that need to be addressed.’

Reflective statement 2: ‘My involvement in the MELD‐B project has allowed me to engage and collaborate with other researchers and contributors in order to produce the workshop, whilst simultaneously expanding my awareness and understanding of public health issues and various contributory factors. I have influenced and contributed to discussions involving parts of the design, structure and delivery of the workshops in regular meetings with the team, using personal experiences with public health research, working with young people, and public presentations. Being able to share ideas, learn from, and work with a collaborative team has made my involvement with this project an enriching experience.’

### 2.2 Stakeholder Invitations

We initially invited all (*n* = 25) stakeholders who had attended our previous stakeholder workshop held in 2023 [11]. We then extended invitations to those who had been invited to the previous workshop but had been unable to attend. Additional stakeholders were identified by the research team, including academics, a public health consultant, policy associates, and public contributors. Further stakeholders were identified through snowball sampling.

The criteria for invitation required that stakeholders had professional expertise or experience in working with children or families. Efforts were made to invite stakeholders who collectively represented the full span of childhood, from pregnancy through to the age of 18.

To support participation by stakeholders who were new to the MELD‐B project, and to remind those who had previously attended the workshop in 2023, invitation emails included a brief overview of the project objectives. This included links to the project website, and a short animated video explaining the MELD‐B research programme. Prior to the workshop, participants were also provided with two summary documents. The first was a one‐page overview of findings from the previous stakeholder workshop, and the second was a one‐page summary of MELD‐B findings on life‐course determinants of health. These materials were intended to provide participants with sufficient background knowledge of the project and its emerging findings, enabling informed contributions to workshop discussions regardless of their prior involvement with MELD‐B.

### 2.3 Conducting the Workshop

The workshop followed a similar format to our previous stakeholder workshop [11]. It began with a short PowerPoint presentation delivered by members of the research team. The presentation summarised findings from the previous stakeholder workshop and demonstrated how stakeholder feedback had been integrated into subsequent MELD‐B research. It also presented key findings from the MELD‐B programme, including research examining the life‐course determinants of MLTC and the potential long‐term impacts of early‐life interventions. This provided context for the workshop discussions and enabled stakeholders to reflect on the project's progress and relevance to current policy and practice. The workshop was held online via Microsoft Teams [20] during normal working hours and lasted 2 h. Stakeholders were then divided into three breakout groups, which were intentionally mixed to ensure a range of professional expertise within each group. Each breakout group was supported by two facilitators. One facilitator led the discussion, and the second took notes and summarised key points for feedback during the full‐group sessions. One public contributor attended the workshop and supported facilitation of both the full‐group discussions and one breakout group.

Each breakout group met twice, with discussions structured around two sets of questions, outlined below.

**Discussion 1:**

- Which current policies have the greatest potential to improve children's health and wellbeing?
- How might changes in the political landscape (e.g., education reforms, benefit caps) affect children's long‐term health outcomes?
- Which policies have the greatest potential to reduce health inequities?
- What research is needed to inform these policies?

**Discussion 2:**

- What is your main takeaway from the MELD‐B research findings?
- Are these findings relevant to your work?
- How can our research findings be made more useful for decision‐making?
- What barriers exist to implementing effective early‐life policies in your area? Can you share any successful interventions we can learn from?

Padlet Inc. [19] was used as an online interactive whiteboard to collate ideas. Stakeholders and facilitators could contribute anonymously during the workshop and after it had concluded. Following each breakout discussions, all stakeholders reconvened in a full‐group session, where facilitators from each breakout group summarised key messages to support a collective discussion.

### 2.4 Identifying Themes and Dissemination

As this was a stakeholder engagement activity rather than a formal qualitative research study, the purpose of the analysis was to identify and narratively synthesise key stakeholder messages rather than undertake a formal qualitative analysis.

Outputs from the Padlet board, breakout room transcripts and full‐group discussions were reviewed together to identify recurring topics, areas of consensus and issues considered important by stakeholders. Initial organisation and summarisation of these materials were supported by an institutional Microsoft M365 Copilot account (GPT‐5 model) [21]. This was used as a supportive tool to assist with organising, synthesising and structuring the qualitative information. Copilot did not generate new content. Copilot functioned solely as an efficiency tool within the analytic workflow, comparable to qualitative software such as NVivo [22], but with enhanced summarisation capabilities. Its use was limited to:

1. Extracting and collating relevant text from the Padlet and meeting transcripts,
2. Assisting with the organisation of themes, and
3. Producing initial summaries of these themes for researcher review.

Themes were subsequently reviewed against the original materials (S.S) and refined through discussion between members of the research team (S.S., S.F. and N.A.), before being circulated to the wider team. All team members, including the public contributors, were invited to contribute to the writing process by reviewing and editing draft outputs. All feedback was considered equally. The final themes represent a synthesis of stakeholder priorities and perspectives arising from the workshop discussions.

### 2.5 Reflexivity

N.A. has a clinical background and is a subject expert in public health, life‐course epidemiology and health inequalities with personal experience of living with a long‐term condition (LTC). S.S. is a postdoctoral research fellow with expertise in life‐course epidemiology with expertise in childhood. S.F. is an academic MLTC subject expert and has experience of caring for people with MLTC in general practice. N.Z. is a lecturer with expertise in life‐course and nutritional epidemiology. EH is a public health registrar and Ph.D. candidate in MLTC research. J.W. is a PPI member with personal experience of living with LTCs. E.P. and R.N. are both young researchers with recent experience of early‐life. R.P. is a public health consultant in a local authority whose portfolio includes healthcare public health. I.C. is a senior public policy associate and a Ph.D. candidate in Nanomaterial Physics. The authors' professional and lived experiences meant the process of designing the workshop was informed by multiple perspectives.

### 2.6 Ethics Statement

This study should be considered as public involvement/contribution in research. The workshop, being stakeholder engagement and not research, did not require ethical approval. Experts by lived experience who took part in our workshop were not research participants. Ethics approval for the overall MELD‐B study, of which this research was a part, was obtained.

## 3 Findings

Fifteen stakeholders from backgrounds including health visiting (*n* = 2), charities (*n* = 1), academics (*n* = 3), healthcare practitioners (*n* = 5) and council employees (*n* = 4), attended the workshop. This reflected a broad mix of expertise across clinical practice, early‐years services, public health and community support. This balanced spread provides strong representation from both service delivery and policy‐focused roles.

The main takes from the workshop can be summarised under two overarching themes— (1) structural barriers and drivers of inequality and, (2) best practice and coordinated early years interventions as highlighted in Figure 2.

**Figure 2:**
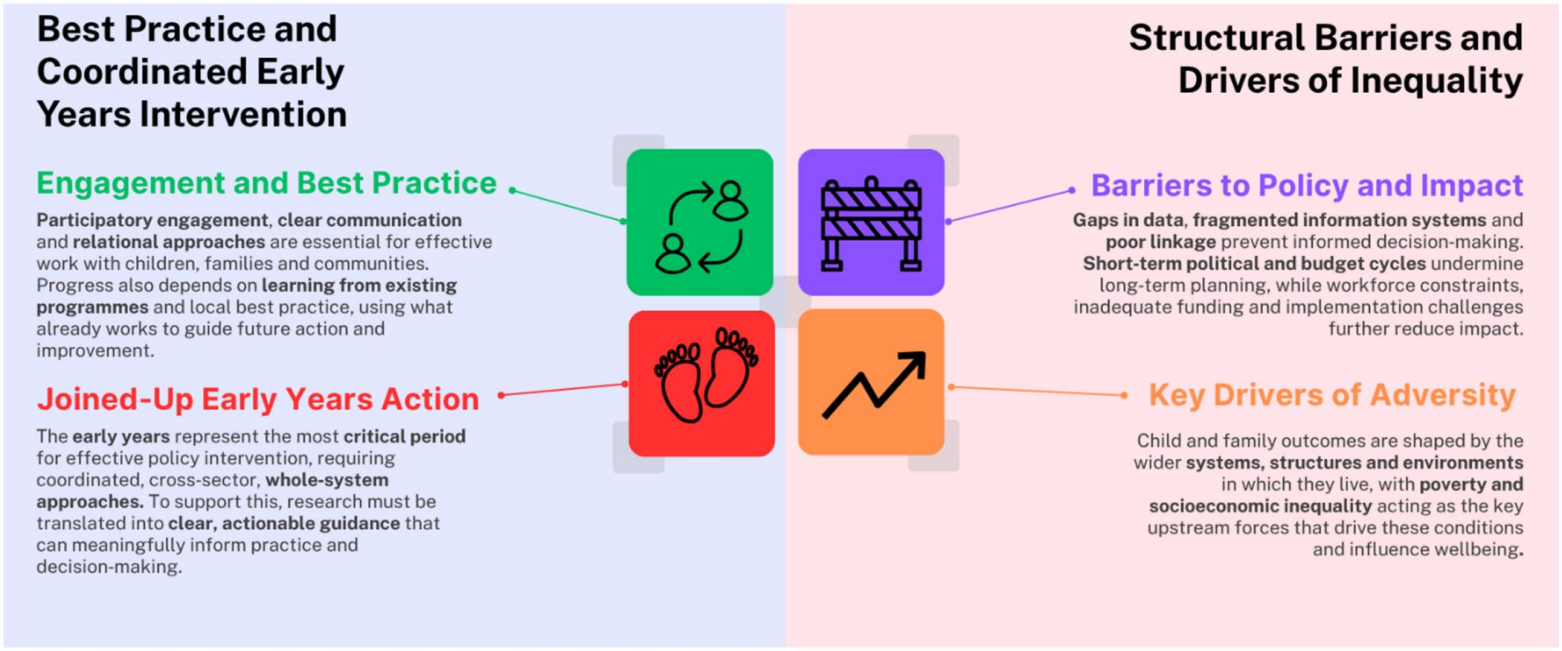
Key themes from the stakeholder workshop.

### 3.1 Structural Barriers and Drivers of Inequality

This section outlines the structural barriers that limit the effectiveness of early‐life policies, as well as the underlying drivers of inequality that shape children's outcomes.

#### 3.1.1 Barriers to Policy and Impact

Stakeholders discussed that more coherent cross‐government child health policy, with shared metrics and aligned incentives, is required to support population‐level health gains. Stakeholders highlighted the need for shared outcomes and improved data infrastructure, greater cross‐system accountability and better aligned incentives, and noted that pressures within the Special Educational Needs and Disability (SEND) system often spill across sectors. They also emphasised that stabilising delivery across electoral cycles is essential for meaningful change. Short political cycles, shifting priorities, and variable funding were seen to undermine impact, while stop–start implementation limits cumulative gains and creates uncertainty. Additional concerns were raised about the lack of a clear national strategy on key issues such as school attendance.

Across the workshop, stakeholders expressed enthusiasm with a broad improvement in potential availability of better data, yet reported significant barriers relating to data access, linkage and quality. They emphasised major data gaps, and the need for richer longitudinal linkages (such as electronic healthcare records) to better model policy impacts. Stakeholders also noted fragmented access to existing datasets and increasing momentum towards local data linkage, including integration of NHS and local authority datasets. Others described difficulties in measuring impact and demonstrating progress, particularly in settings with constrained budgets and short time horizons.

A consistent barrier identified by stakeholders was the mismatch between the long‐term impacts of early‐life interventions and the short‐term political or commissioning cycles that shape decision making. Stakeholders explained that making the long‐term case for early‐life investment is essential, given the contrast between 1 and 3 year budgeting cycles and benefits that unfold over two to three decades. They also emphasised the political importance of framing evidence in ways that resonate with policymakers, particularly in relation to prevention and cost‐saving narratives, and expressed interest in modelling the long‐term impacts of national policies to articulate benefits more clearly under resource constraints.

Finally, stakeholders described broader structural barriers that hinder the delivery of effective early‐life policies. These included funding cuts, short‐termism and challenges engaging schools and early years settings because of workforce pressures. They also highlighted constraints around real‐world feasibility, such as pregnancy not always being an optimal time for interventions and the limited generalisability of some trial evidence, as well as concerns about data access barriers, political ‘spin’ and system complexity in constrained economic environments.

#### 3.1.2 Key Drivers of Adversity

Stakeholders consistently framed income and socioeconomic factors as key upstream drivers of inequality shaping almost every other outcome. Examples discussed to improve socioeconomic outcomes included ending the two child benefit cap limit (a cap meaning families can only receive child benefits for their first two children), increasing Healthy Start vouchers (a prepaid card to help low‐income families buy healthy food) and implementing broader child poverty strategies. Stakeholders also highlighted the role of benefit policy and food security in determining nutrition and wellbeing, and emphasised poverty and wider socioeconomic conditions as underlying factors influencing attendance, learning, access to services and daily decision making.

### 3.2 Best Practice and Coordinated Early Years Interventions

This section outlines stakeholders' perspectives on how best practice and coordinated approaches can strengthen early years policies.

#### 3.2.1 Engagement and Best Practice

Stakeholders discussed best practices for addressing unhealthy environments and emphasised that system‐level environmental levers were viewed as more effective and equitable than individual‐level interventions. Examples included expanding the sugar tax, improving food labelling and introducing zoning restrictions for fast food restaurants, shops and takeaways around schools. Stakeholders also highlighted the need for better designed play parks and environments that support physical activity, and recognised that poor‐quality food environments are disproportionately concentrated in areas affected by poverty.

In terms of wider engagement, stakeholders stressed the importance of co‐production and the involvement of those directly affected, noting that children's and parents' priorities are often under‐represented. A shift towards participatory design was seen as a way to enhance the relevance, uptake and equity of policy interventions. Stakeholders also emphasised the need for better real‐world evidence infrastructure to enable policymakers to act confidently and consistently.

With regard to best practice, stakeholders valued ongoing research by academic partners but expressed a strong desire for more applied interpretation. This included clearer implications for programme design, commissioning and local resource allocation, as well as findings that translate directly into guidance on ‘what to do’ rather than solely identifying associations. Stakeholders also called for practical decision support tools to help target resources in realistic ways that go beyond traditional measures such as the Index of Multiple Deprivation (IMD). They noted the importance of aligning research outputs more closely with real‐time decision cycles and commissioning priorities, particularly for local authorities.

Across the workshop, stakeholders highlighted that how messages or support are communicated matters greatly, noting that parent–child interactions remain a key pathway for engagement and good practice. They also stressed the value of presenting findings using resilience and protective factors, not only risks, as this framing is often more engaging for practitioners and families. In addition, stakeholders reflected on cultural expectations and the challenges of engaging schools and early years settings in the context of workforce pressures.

Stakeholders saw value in building on existing interventions rather than reinventing programmes, and expressed interest in research that helps identify which interventions scale most effectively and what can be learned from exemplars of good practice at both local and national levels. Examples included LifeLab, My Happy Mind and the Healthy Early Years Programme, which were described as strong local assets with evaluation evidence of growing uptake. Sure Start was cited as a benchmark with the rich national evaluation evidence, and stakeholders noted that new Family Hubs will generate substantial data in the coming years. Stakeholders also emphasised strengths in emerging cross‐sector collaborations and in opportunities for data‐enabled targeting, such as maternal–child linkage.

#### 3.2.2 Joined‐Up Early Years Action

One of the overarching themes across the workshop was the importance of investing in high‐quality, inclusive early years policies. Stakeholders viewed this as one of the most powerful opportunities for improving population health across the life course, identifying the early years as the period in which policy action can deliver the greatest long‐term impact on health, wellbeing and inequalities. Stakeholders called for strong and stable early years programmes with sustained investment in Family Hubs, for expanding access to and equity in childcare particularly for children with SEND, and for prevention efforts to begin much earlier than school age supported by consistent early messaging.

Stakeholders also emphasised the need to develop a clearer understanding of how whole‐system working operates in practice, including interactions across health, education and social care systems. They noted that the concept of ‘system change’ requires clearer definition to ensure it is meaningful and actionable within policy and service delivery contexts.

## 4 Discussion

Through this workshop, we heard from stakeholders from various backgrounds including health visiting, charities, academia, healthcare practitioners and council employees about the barriers to early‐life policies as well as areas of best practice to guide future action.

Stakeholders highlighted challenges such as fragmented policy systems, short‐term political cycles, data gaps and difficulties in linking evidence to real‐world decision making. They also emphasised that socioeconomic conditions and poverty act as major upstream determinants that influence a wide range of outcomes including health, learning, access to services and daily decision making. Together, these structural constraints and drivers of adversity create significant obstacles to achieving meaningful and sustained improvements in children's health across the life course.

In contrast, stakeholders emphasised the importance of creating healthy and supportive environments through system‐level action, meaningful co‐production and clearer, more actionable research translation. They also highlighted the need for strong, inclusive early years policies, coordinated working across sectors and investment in early intervention to maximise long‐term impact on children's health, wellbeing and inequalities.

The stakeholder discussions reflect systemic issues such as fragmented policy environments, short political and commissioning cycles, entrenched socioeconomic inequalities and persistent data gaps. These challenges are situated within the wider UK context. For example, UCL [23] recently published a report highlighting the persistence and evolution of structural inequalities in the United Kingdom, emphasising multiple and intersecting forms of socioeconomic disadvantage, including unequal access to resources, opportunities and the high‐quality data required for effective policy responses. Similarly, Thompson et al. [24] demonstrate how poor data quality can hinder service decision‐making, negatively impact child behavioural health and wellbeing, increase unnecessary expenditure of time and resources, undermine the validity of research and slow the policymaking process. Together, these systemic constraints reinforce many of the barriers identified by stakeholders in this workshop and illustrate the broader structural context within which early‐life policy operate.

The stakeholder discussions support the wider public health literature on the importance of early‐years investment for outcomes across the life course. Their discussions support theoretical perspectives such as life course frameworks [25, 26], the ‘long arm of childhood’ [27, 28, 29, 30] and life course health development frameworks [25, 31] that all demonstrate how childhood exposures can have long‐term associations with demographic and health outcomes in adulthood. From a UK political perspective, stakeholder discussions support continued investment in early‐years policies, linking to UK government reports including *The First 1000 Days of Life* [32], *The Best Start for Life* [33] and *Giving Every Child the Best Start in Life* [34]. Finally, from a research perspective stakeholder discussions support a large body of research that has consistently found a range of early‐life factors to be important for health across the adult life course [35, 36, 37].

Stakeholders described two specific tensions between research and policy. First, they noted that long‐term life course research such as the MELD‐B project, which explores outcomes and benefits unfolding up to 35 years after early‐life exposures, do not align with the short‐term political cycles that shape policy priorities. This mismatch creates a divide between political focus and the research agenda. Stakeholders suggested that one way to help bridge this gap is to emphasise intermediate outcomes, such as those identified at the end of childhood that lie on the causal pathway to adult health, as outlined in the previous stakeholder workshop [11]. Stakeholders highlighted temporal tensions related to the pace of academic research itself, noting that the time required to generate robust evidence often does not align with the real‐time short policy cycles and commissioning priorities faced by local authorities. These tensions underscore the structural challenges of ensuring that academic evidence can meaningfully inform policy within the timeframes in which decisions must be made.

Stakeholders identified several priorities for strengthening the translation of academic research into policy and practice. They emphasised the need for research outputs to be clearer and to provide more practical guidance to support implementation, alongside more applied interpretation of findings to ensure their relevance for real world decision making. However, it is important to note that in some contexts, particularly when assessing early‐life interventions with outcomes that emerge in midlife, higher‐quality evidence such as randomised controlled trials may not be feasible. As a result, policy decisions may need to draw on evidence lower down the traditional hierarchy of evidence. Stakeholders also highlighted the importance of developing research tools and outputs that are feasible to use within local constraints and commissioning cycles, and noted the value of framing evidence around protective factors rather than focusing solely on risk. In addition, stakeholders stressed the need to expand co‐produced research involving people with lived experience, particularly parents, to ensure future work reflects the priorities and perspectives of those most directly affected. Collectively, these priorities underline areas to which researchers should give careful consideration when conducting work intended to inform early‐life policy and practice.

### 4.1 Strengths and Limitations

A strength of this activity was the ability to engage with a diverse group of stakeholders whose roles covered various time points across childhood. We integrated the perspectives of three public contributors that included two young researchers into the design of the workshop to ensure their views influenced the process, and we involved a patient and public contributor at every stage of the workshop design and conduct cycle.

Despite invitations being sent to all stakeholders who attended the previous workshop, along with additional identified stakeholders, the number who attended this workshop was lower than before, potentially reducing continuity across workshops. A further limitation was the relatively small number of stakeholders overall, combined with the constraint that the workshop could only be conducted once. Our stakeholder group was also predominantly based in the South East region of the UK, reflecting the geographical connections of the research team. As a result, the perspectives represented may not fully reflect those of other regions across the country, which may have different priorities.

While the workshop was collaboratively developed with public contributors throughout its design, delivery and dissemination, stakeholder involvement was primarily consultative. Stakeholders contributed expert perspectives through structured discussions. Therefore, the stakeholder engagement component should be interpreted as consultation informed by a collaborative design rather than stakeholder co‐production itself. Finally, although the workshop was framed around MLTC, discussions often focused more broadly on children's health and wellbeing. As a result, the findings primarily reflect stakeholder perspectives on early‐life determinants of health rather than specific long‐term conditions. However, these broader health outcomes remain relevant to MLTC research, as many of the factors identified are upstream determinants that may influence trajectories towards the development of MLTC across the life course.

## 5 Conclusions

Early‐life policies are important for supporting children and their families at an early stage to mitigate risk factors and strengthen protective factors for health and wellbeing across the lifecourse, thereby reducing the risk of MLTC later in life. Our workshop demonstrated the value of collaborative engagement in identifying both the structural barriers and the practical opportunities for strengthening early‐life policy. Stakeholders described how structural constraints, including fragmented systems, short‐term funding cycles and socioeconomic inequalities, can limit the implementation and effectiveness of policies intended to improve children's health and wellbeing. These factors are relevant to MLTC prevention because they influence early‐life exposures and opportunities that shape health trajectories across the life course. However, stakeholders also highlighted that joined‐up investment in inclusive early‐years policies, coordinated working across sectors and sustained early intervention, alongside learning from and building on existing successful programmes, offers potential to improve children's health and wellbeing, reduce inequalities and support the long‐term prevention of MLTC.

## Author Contributions

**Sebastian Stannard:** conceptualisation, writing – original draft, writing – review and editing, methodology, visualisation, investigation, project administration. **Nida Ziauddeen:** conceptualisation, investigation, writing – original draft, writing – review and editing, methodology. **Robin Poole:** writing – review and editing, conceptualisation, investigation, writing – original draft. **I. Cook:** conceptualisation, investigation, writing – original draft, writing – review and editing, methodology. **Emilia Holland:** writing – original draft, writing – review and editing, investigation. **Erin Panes:** methodology, writing – original draft, writing – review and editing, visualisation, investigation. **Reet Nagra:** investigation, methodology, writing – review and editing, writing – original draft, visualisation. **Jack Welch:** investigation, writing – original draft, writing – review and editing, visualisation, methodology. **Simon DS Fraser:** funding acquisition, writing – original draft, writing – review and editing, investigation, conceptualisation. **Nireen A Alwan:** methodology, conceptualisation, investigation, funding acquisition, writing – original draft, writing – review and editing, visualisation.

## Conflicts of Interest

The authors declare no conflicts of interest.

## Data Availability

Data sharing is not applicable to this article as no datasets were generated or analysed during the current study.
